# Using stable isotopes to estimate travel times in a data‐sparse Arctic catchment: Challenges and possible solutions

**DOI:** 10.1002/hyp.13146

**Published:** 2018-05-29

**Authors:** Doerthe Tetzlaff, Thea Piovano, Pertti Ala‐Aho, Aaron Smith, Sean K. Carey, Philip Marsh, Philip A. Wookey, Lorna E. Street, Chris Soulsby

**Affiliations:** ^1^ Northern Rivers Institute, School of Geosciences University of Aberdeen Aberdeen AB24 3UE United Kingdom; ^2^ IGB Leibniz Institute of Freshwater Ecology and Inland Fisheries Berlin Germany; ^3^ Department of Geography Humboldt University Berlin Berlin Germany; ^4^ School of Geography and Earth Sciences McMaster University Hamilton Ontario Canada; ^5^ Dept. of Geography and Cold Regions Research Centre Wilfrid Laurier University Waterloo Canada; ^6^ Faculty of Natural Sciences, Biological & Environmental Sciences University of Stirling Stirling FK9 4LA United Kingdom; ^7^ School of GeoSciences University of Edinburgh Edinburgh United Kingdom

**Keywords:** active layer, Arctic headwaters, isotopes, permafrost, transit times

## Abstract

Use of isotopes to quantify the temporal dynamics of the transformation of precipitation into run‐off has revealed fundamental new insights into catchment flow paths and mixing processes that influence biogeochemical transport. However, catchments underlain by permafrost have received little attention in isotope‐based studies, despite their global importance in terms of rapid environmental change. These high‐latitude regions offer limited access for data collection during critical periods (e.g., early phases of snowmelt). Additionally, spatio‐temporal variable freeze–thaw cycles, together with the development of an active layer, have a time variant influence on catchment hydrology. All of these characteristics make the application of traditional transit time estimation approaches challenging. We describe an isotope‐based study undertaken to provide a preliminary assessment of travel times at Siksik Creek in the western Canadian Arctic. We adopted a model–data fusion approach to estimate the volumes and isotopic characteristics of snowpack and meltwater. Using samples collected in the spring/summer, we characterize the isotopic composition of summer rainfall, melt from snow, soil water, and stream water. In addition, soil moisture dynamics and the temporal evolution of the active layer profile were monitored. First approximations of transit times were estimated for soil and streamwater compositions using lumped convolution integral models and temporally variable inputs including snowmelt, ice thaw, and summer rainfall. Comparing transit time estimates using a variety of inputs revealed that transit time was best estimated using all available inflows (i.e., snowmelt, soil ice thaw, and rainfall). Early spring transit times were short, dominated by snowmelt and soil ice thaw and limited catchment storage when soils are predominantly frozen. However, significant and increasing mixing with water in the active layer during the summer resulted in more damped steam water variation and longer mean travel times (~1.5 years). The study has also highlighted key data needs to better constrain travel time estimates in permafrost catchments.

## 1 INTRODUCTION

Our understanding of water sources, flow paths, and run‐off generation processes remains dominated by studies conducted in humid temperate regions, where precipitation is predominantly rain and exceeds evapotranspiration, and run‐off generation is largely influenced by subsurface processes (Tetzlaff et al., 2015). However, relatively understudied, data‐sparse Arctic environments are currently experiencing some of the most rapid rates of environmental changes as a consequence of global warming, with limited benchmarks against which to assess the implications (Adam, Hamlet, & Lettenmaier, 2009; Bring et al., 2016; DeBeer, Wheater, Carey, & Chun, 2016; Walvoord & Kurylyk, 2016; White et al., 2007). Changes in air temperatures influence cryogenic processes that play a key role in energy and water balances in Arctic regions (Woo, Kane, Carey, & Yang, 2008). Aside from prolonged snow cover, spring melt, and autumn freeze (DeWalle & Rango, 2008; Hinzman, Kane, Benson, & Everett, 1996), the most notable features influencing the hydrology of Arctic headwaters is the presence of permafrost, which is ground that remains at or below 0 °C for two or more consecutive years. Although permafrost depth and distribution is variable across circumpolar regions, it has a strong influence on run‐off pathways as it effectively acts as an aquitard (Woo, 1986). Capturing these processes in hydrological models is challenging. Permafrost thaw rates are accelerating and expected to have cascading effects on Arctic ecosystems, river flow regimes, and associated biogeochemical interactions (Bring et al., 2016; Frey & McClelland, 2009; Lafrenière & Lamoureux, 2013; Lessels, Tetzlaff, Carey, Smith, & Soulsby, 2015; Pokrovsky et al., 2015; Walvoord & Striegl, 2007). Despite these rapid changes, there are still limited studies in Arctic headwater catchments, and long‐term monitoring sites are declining (Laudon et al., 2017). However, such studies are critical to inform policymakers on the local hydrological impacts of environmental change and how these propagate to larger river systems.

Environments in Arctic regions are complex and often have a strong legacy of glaciation, widespread organic soils, and heterogeneous unconsolidated glacial materials affecting water flow paths and storage (e.g., Paquette, Fortier, & Vincent, 2017; Quinton & Marsh, 1998; Rushlow & Godsey, 2017). Continuous permafrost confines flow paths to the surface and near‐surface zone, termed the *active layer* (i.e., the transient zone of seasonal freeze and thaw). Catchments with continuous permafrost are usually characterized by flashy hydrograph responses as snowmelt and near‐surface drainage of the active layer dominates annual run‐off contributions. In these catchments, baseflow is limited, and there is typically a cessation of flows during freezeback as deeper flow pathways are absent (Woo, 2012). The exception is where unfrozen taliks allow for deeper groundwater to interact with the surface (Michel & Van Everdingen, 1994). Recent work has highlighted the influence of thawing permafrost on activating deeper flow paths, resulting in extended recessions and increasing autumn and winter flows (Smith, Pavelsky, MacDonald, Shiklomanov, & Lammers, 2007; St. Jacques & Sauchyn, 2009; Walvoord, Voss, & Wellman, 2012).

Subsurface complexities, together with the remoteness and logistical difficulties associated with access and data collection in many Arctic headwater catchments, limits empirical studies and process understanding. This makes environmental tracers, particularly stable isotopes, potentially useful tools for hydrological monitoring. Tracers provide integrated insight into the hydrological functioning of catchments and have been used previously to assess water sources and flow paths in Arctic and permafrost settings (Ala‐aho, Soulsby, et al., 2017; Blaen, Hannah, Brown, & Milner, 2014; Lamhonwah, Lafrenière, Lamoureux, & Wolfe, 2017; Obradovic & Sklash, 1986; Song et al., 2017; Yi et al., 2012). In addition to their capacity to quantify water provenance, flow paths, and transit times, tracer studies provide insights for calibration and testing more detailed conceptual and numerical models at different spatial scales (Ala‐aho, Tetzlaff, McNamara, Laudon, & Soulsby, 2017; Birkel, Soulsby, & Tetzlaff, 2011; Soulsby et al., 2015; Stadnyk, Delavau, Kouwen, & Edwards, 2013; van Huijgevoort, Tetzlaff, Sutanudjaja, & Soulsby, 2016).

The presence of permafrost and snowmelt poses challenges and opportunities to adequate sample collection to facilitate the application and interpretation of tracer‐based methodologies developed in more temperate catchments (Tetzlaff et al., 2015). The depleted isotopic composition of snow creates a traceable hydrological signal at freshet, which has been used to understand run‐off generation processes (Carey & Quinton, 2004; Hayashi, Quinton, Pietroniro, & Gibson, 2004; Laudon, Seibert, Köhler, & Bishop, 2004). In an Alaskan catchment underlain by continuous permafrost, McNamara, Kane, and Hinzman (1997) concluded that the spring freshet was supplied largely by new meltwater inputs, with pre‐event water dominating stormflow hydrographs generated by summer rainfall. However, such large event water contributions during snowmelt are inconsistent with estimates of significant pre‐event water contributions to streamflow during snowmelt in other permafrost landscapes (e.g., Ala‐aho et al., 2018; Carey, Boucher, & Duarte, 2013; Gibson, Edwards, & Prowse, 1993; Obradovic & Sklash, 1986). In a discontinuous permafrost alpine catchment in Yukon, Canada, Carey and Quinton (2004) assessed the dynamics of water sources and flow paths during the critical snowmelt period. There, run‐off contributing areas were defined by the presence of permafrost, and the development of the active layer on permafrost‐influenced slopes resulted in a gradual decrease in meltwater contribution to streamflow during snowmelt and streamflow was dominated by pre‐event water by the end of melt. This suggests that the pre‐event water component in streamflow from permafrost catchments at the start of snowmelt is most likely water held in the often widespread organic soil that mantles the slopes (Carey et al., 2013; Carey & Quinton, 2004; McNamara et al., 1997). The major seasonal shift in Arctic catchments, together with ongoing, spatially distributed patterns of freeze–thaw over different timescales, represent a significant challenge for using isotopes in hydrological assessment as sampling ideally needs to encompass the entire period between the start of spring melt and the autumn freeze.

Stable isotopes can be used to estimate water transit or travel times (TTs), defined as the elapsed time between water entry to, and exit from, a catchment as stream discharge at the outlet. TTs represent the length of time needed for a parcel of water takes to traverse storage from input to output. The simplest, traditional method for estimating TTs uses lumped parameter inverse modelling of isotopes assuming time invariant TT distributions and has a long history in cold regions (e.g., Dinçer, Payne, Florkowski, Martinec, & Tongiorgi, 1970; Lyon et al., 2010; Maloszewski, Rauert, Stichler, & Herrmann, 1983; Rodhe, Nyberg, & Bishop, 1996). For example, in a study at 16 sites in northern Sweden, Lyon et al. (2010) found that the mean TT associated with snowmelt water release varied from between 20 and 180 days, depending on landscape factors such as percentage of wetland areas and average site gradient. However, in permafrost environments, this requires that the models be driven by the time variant input signals from snow and soil thaw which are difficult to measure. Furthermore, impervious boundaries to vertical infiltration of water during snowmelt periods developed through (discontinuous) permafrost alter the flow paths of water, influencing TTs in a time varying way (Walvoord et al., 2012). Lyon et al. (2010) suggested that potential thaw of these ice layers due to climate change could increase mean TTs at the catchment scale by 20% to 45% assuming different soil and till thicknesses. Despite the global significance of the hydrological and biogeochemical implications of such increased thaw and TTs in permafrost regions, we have remarkably limited data and tools to benchmark future change.

Here, we present results from a stable isotope study in a small headwater catchment, Siksik Creek, in the western Canadian Arctic. Previous work in Siksik Creek has shown that interhummock channels draining thawing surface horizons of organic peat soils result in rapid run‐off generation, and this is the greatest contribution to the stream network (Quinton & Marsh, 1998, 1999). The overall aim of this paper was to use water stable isotopes to help identify the sources of run‐off and make a first approximation of the TT of this water through the catchment. We use the study as an exemplar of some of the challenges and potential solutions to TT analysis in such catchments. Within this context, the specific objectives were to

- use stable isotope data sampled in precipitation, snowmelt, soil water, and surface water to investigate dynamics of water sources and flow paths in an Arctic headwater catchment;
- develop an appropriate framework of model–data fusion to estimate the isotope composition of snowmelt and thawing soil water; and
- make a preliminary estimation of TTs with focus on the transition period between late snowmelt and soil thaw.

From this, we will discuss the future challenges and data needs for stable isotope and TT applications in data‐sparse Arctic regions with an outlook to guide future work at a time of marked climate change.

## 2 DATA AND METHODS

### 2.1 Study site

**Figure 1:**
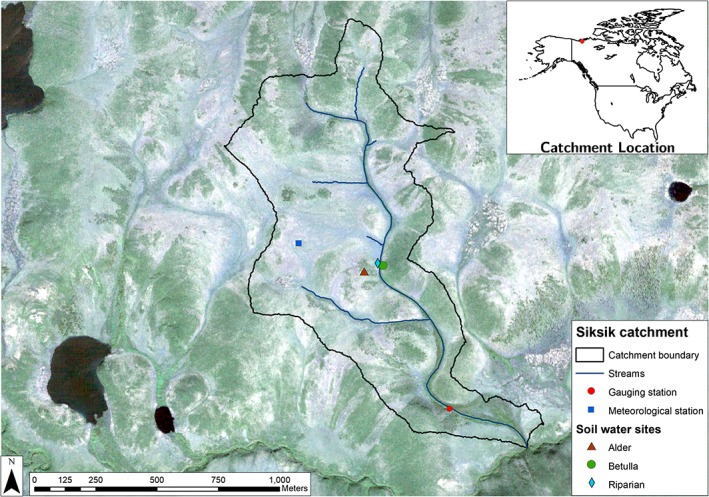
Map and location of the Siksik Creek catchment (NWT, Canada). The map also shows stream and location of gauging station, meteorological station, and locations of the soil water sampling sites (alder, *Betula*, and riparian)

The Siksik Creek catchment (0.92 km^2^) is a subcatchment of Trail Valley Creek, located approximately 45 km NNE of Inuvik in Northwest Territories, Canada (68°44′17 N, 133°26′26″W). This long‐term experimental catchment has elevation ranges from 50 to 100 m a.s.l. (Figure 1). Siksik is located in the continuous permafrost zone of the western Canadian Arctic (Heginbottom & Radburn, 1992): on the border of the subarctic (Dfc) and tundra (Et) climates, according to the Köppen classification (Peel, Finlayson, & McMahon, 2007). Mean annual air temperature at Inuvik climate station is −8.2 °C, summers are short and cool (12 °C for June–August), whereas winters are long and cold (−26 °C for December–February; 1981–2010; Environment Canada, 2010). Precipitation averages 241 mm, with approximately 66% occurring as snowfall and the remainder as summer rains (Environment Canada, 2010).

Summer active layer depths range from 0.4 to 0.8 m, whereas maximum permafrost thickness varies from 350 to 575 m (Natural Resources Canada, 1995). The ground surface is dominated by periglacial features: thermokarst, ice‐wedge polygons, and mineral earth hummocks that can be bare or overlain with a thin layer of lichen (Marsh, Quinton, & Pomeroy, 1995). Interhummock areas consist of peat (0.2–0.5 m depth; Quinton & Marsh, 1999) and are characterized by a hydraulic conductivity that varies between 10^−3^ and 10^−6^ m s^−1^, whereas the conductivity in mineral hummocks is less than 10^−7^ m s^−1^ (Marsh et al., 1995).

The vegetation of the area consists predominantly of ericaceous shrubs, sedges (*Eriophorum* and *Carex* spp.), bryophytes, and lichens, with patches of tall shrubs on hillslopes (*Alnus viridis* and *Betula glandulosa*). The riparian zone is characterized by *B. glandulosa* and *Salix* spp.

The hydrology of Siksik Creek is dominated by spring snowmelt and freshet, accounting for over 90% of annual stream discharge. Stream discharge is typically characterized by few peaks in late spring and is low during the rest of the summer period, with modest responses to rain events (Marsh et al., 1995). The dominant mechanism of run‐off is shallow subsurface flow in the active layer through interhummock troughs as outlined by Quinton and Marsh (1999).

### 2.2 Monitoring

Hydrometric measurements and stable water isotopes samples were collected at the study site in the spring and summer of 2014. Event‐based rainfall samples were collected with an autosampler (with paraffin added to sample bottles to prevent evaporation), emptied at daily resolution at Siksik Creek, and supplemented by rainfall samples at the nearest town (Inuvik) when local samples were not available. Because of access difficulties during winter, snowfall was not sampled. Snowmelt water samples were collected from the late‐lying remaining snowpack in the spring/summer. Daily streamwater samples were collected with an autosampler at the gauging station (Figure 1). However, due to technical problems, the sampler was not working for a period between late June and early July. Mobile soil water samples were collected at 10‐cm depth at three different sites (riparian, alder [*A. viridis*]) and dwarf birch (*B. glandulosa*) sites, with increasing distance from the stream and decreasing depth of an organic O horizon from >0.4‐ to 0.2‐m depth for the riparian and birch site, (Figure 1) on 13 occasions, using MacroRhizon suction cups (MacroRhizon by Rhizosphere Research Products, Wageningen, Netherlands). All water samples were analysed for deuterium (δ^2^H) and oxygen‐18 (δ^18^O) compositions using an off‐axis integrated cavity output spectroscopy (Triple Water‐Vapor Isotope Analyzer TWIA‐45‐EP, Model: 912‐0032‐0000, Serial: 14‐0038, Manufactured: 03/2014, Los Gatos Research, Inc., San Jose, USA) running in liquid mode with a precision of ±0.4‰ for δ^2^H and ±0.1‰ for δ^18^O as given by the manufacturer. Values are expressed in delta per mil (‰) relative to the Vienna Standard Mean Ocean Water standard.

Soil temperature (T_soil_) and volumetric water content (VWC) were also measured hourly at the three soil water sampling locations. The VWC of soils was monitored at 5‐cm depth using a HOBO ECH2O soil moisture probe (Onset Inc, Pocasset, MA, USA). Soil temperatures were logged using a datalogger (CR800, Campbell Scientific, Logan, USA) connected to a 32‐channel relay multiplexer (AM16/32B, Campbell Scientific, Logan, UK). Thaw depth was measured with steel rods on four occasions at each location. Stream stage was recorded with a pressure transducer at the outlet of the catchment (Figure 1). Discharge was derived from a stage–discharge rating curve, regularly updated throughout the study period. The freshet started in late May, but deep snow beds remained until mid‐June, precluding access to the stream gauging station and measurements during early freshet.

Daily climate data, with the exception of shortwave radiation, were measured by Environment Canada (http://climate.weather.gc.ca) at the Trail Valley Creek station. Shortwave radiation was obtained from the global atmospheric reanalysis ERA‐Interim, provided by the European Centre for Medium‐Range Weather Forecasts (ECMWF, http://www.ecmwf.int/en/research/climate-reanalysis/era-interim).

### 2.3 Hydrological modelling and MTT estimation

**Figure 2:**
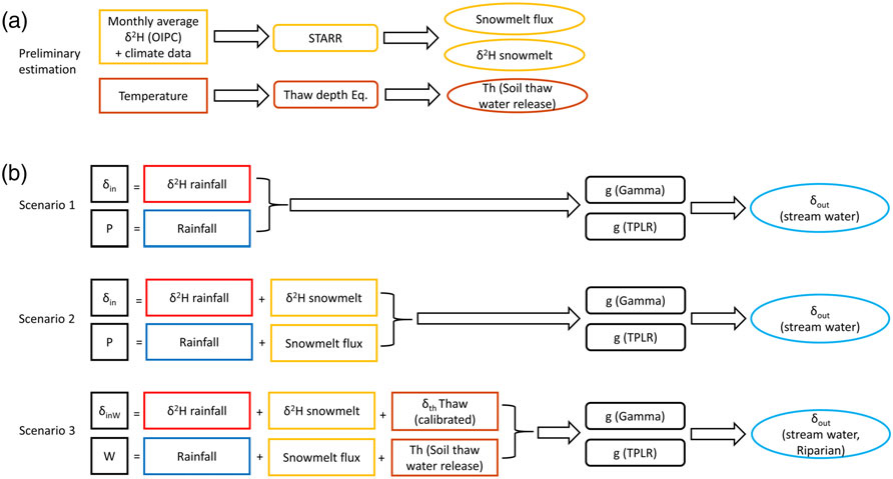
Major methodological steps, summarized as input, applied models, and resulting output in (a) preliminary estimations and (b) different scenarios of integral convolution model. Output of (a) became some of the input in (b). *δ*_*in*_, *δ*_*inW*_, *δ*_*out*_, *δ*_*th*_, *P*, *W*, and *g* relate to Equations (3), (4), and (5). STARR = spatially distributed tracer‐aided rainfall–run‐off model; TPLR = two parallel linear reservoirs

Given the relatively sparse data set compared with more accessible study sites, we used model–data fusion to understand better the hydrological fluxes, isotope dynamics, and TTs at the site (Figure 2). To estimate the isotope composition of snowmelt in late spring (prior to site access), we applied a novel, spatially distributed model developed by Ala‐aho, Tetzlaff, McNamara, Laudon, Kormos, et al. (2017). The model simulates snowpack dynamics (accumulation and melt) with process‐based energy balance equations and isotope compositions in the snowpack and snowmelt run‐off. The simulation routines are based on the assumption of complete isotope mixing in the snowpack and incorporate snowpack sublimation and time‐variable isotopic fractionation of snowmelt. Sublimation and time‐variable melt fractionation processes are important for tracer‐aided studies (Schmieder et al., 2016; Taylor, Feng, Williams, & McNamara, 2002). The model outputs are spatially distributed snowmelt flux and isotopic compositions. Full details of model equations, functionality, and discussion of assumptions and uncertainties are given in Ala‐aho, Tetzlaff, McNamara, Laudon, Kormos, et al. (2017). The snowpack isotope model has been successfully coupled with the spatially distributed, tracer‐aided rainfall–run‐off model STARR (spatially distributed tracer‐aided rainfall–run‐off model; van Huijgevoort et al., 2016) to simulate the isotope ratios of streamflow in a range of northern snowmelt influenced catchments (Ala‐aho, Soulsby, et al., 2017).

Snowpack dynamics and corresponding isotopic compositions were simulated for 2013 (spin‐up period) and 2014 using the snow isotope model. Daily meteorological data (precipitation, relative humidity, air temperature, wind speed, and global short wave radiation) and topographic data (digital elevation model of the catchment, cell size 50 × 50 m) were used as hydrological model inputs. In the absence of comprehensive isotopic sampling during the winter, we used the monthly average precipitation (snowfall) isotope composition estimated from the Online Isotope Precipitation Calculator (OIPC; Bowen, 2017). OIPC estimates precipitation using catchment latitude, longitude, and mean elevation. All days in a given month were assigned the monthly average isotope composition. Suitability of the OIPC precipitation estimates was verified with precipitation samples collected from Siksik and Inuvik.

**Figure 3:**
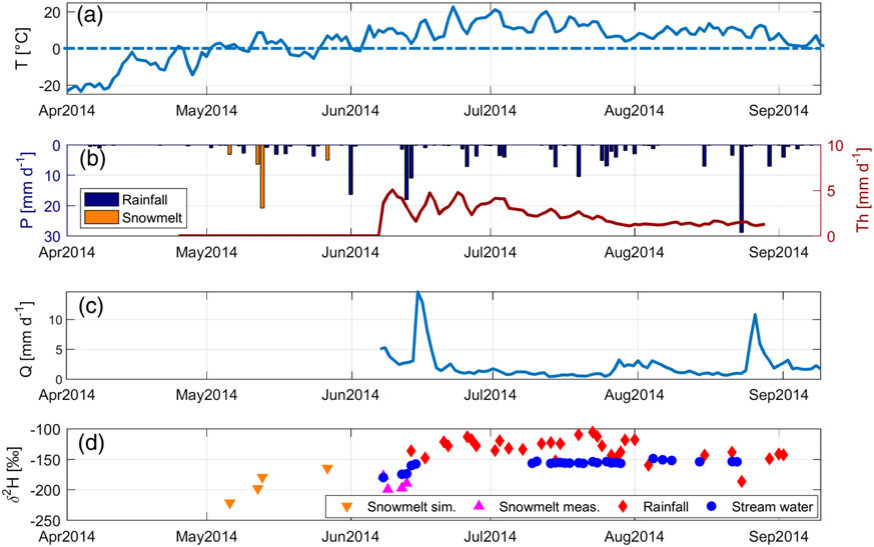
Time series of (a) temperature T (°C); (b) precipitation P (mm; rainfall and estimated snowmelt) and estimated soil thaw water release Th (mm day^−1^); (c) discharge Q (mm day^−1^); and (d) δ^2^H (‰) in snowmelt (both simulated with spatially distributed tracer‐aided rainfall–run‐off model and measured), rainfall samples, and streamwater samples

In the absence of direct snow depth or water equivalent measurements or representative nearby stations, timing of snow ablation was estimated using Landsat satellite imagery (four images without cloud contamination: May 4, May 10, June 4, and June 11, 2014) for snow cover extent. The satellite data suggested that snowmelt initiated after May 4 and had completed by June 4, except for a few late‐lying snow patches. The model was calibrated to match the ablation timing by varying parameters for snowfall under‐catch correction coefficients (influencing the amount of accumulated snow) and snow albedo reduction for aging snow (influencing the rate of snowmelt). Snowmelt and sublimation fractionation parameters were assessed by comparing snowmelt isotopic simulations to snowmelt samples from late‐lying snowpacks. Because timing of the simulated snowmelt was different (earlier) than the sampling of the late‐lying snowpacks (see Figure 3), we could only calibrate the snow isotope model to the range and central value of the observed snowmelt isotopes. Calibration was conducted with the trial and error method until a satisfactory agreement was found between both constraining “soft” calibration datasets and simulated timing and isotope composition of snowmelt.

The freeze–thaw dynamics of the active layer result in an additional source of water for mixing and streamflow generation from previously frozen soil water, and estimating the water release during thawing of the active layer was an essential step in the modelling and TT estimates (Figure 2). As only few direct measurements of thaw depth during the study period were available (four occasions), the dynamics of the active layer were simulated. Freeze–thaw depths of the active layer were estimated using an approximation of the Stefan equation (Hinkel & Nicholas, 1995):

(1) $$z=b{\left(\text{ADDT}\right)}^{0.5},$$

where *z* is the thaw depth (m), *b* describes the thermal structure of the ground and rate of thaw progression, and *ADDT* is the accumulated day degree temperature (°C; i.e., the sum of average daily temperatures above 0 °C). Soil temperature at the Riparian site was used to estimate the ADDT. We used the measured soil temperature instead of air temperature, because it better represented the thermal regime in the thawing soil. We also tried to use air temperature during model testing, but that resulted in too early initiation of soil thaw and too deep thaw depths when compared with our thaw depth data. Thaw depth measurements during the study period were used to calibrate Equation (1). A variable representing the soil thaw water release (Th) was introduced to estimate the flux from the thawing active layer. The progression of the soil thaw water release was estimated using

(2) $$\mathrm{Th}\left(t_{i}\right)=\phi \left(z\left(t_{i}\right)-z\left(t_{i-1}\right)\right),$$

where Th(t_i_) is the thaw water release at day t_i_ (mm day^−1^), ϕ is the unfrozen drainable porosity, z(t_i_) and z(t_i‐1_) are the thaw depths at day t_i_ and the preceding day (t_i−1_). In the absence of measured porosity data, we used literature values to estimate the amount of water that can be released by thawing soil. We considered separately the organic layers (depth 0.05–0.35 m) and the lower mineral soil (depth 0.35–0.45 m). Total porosity of organic soil is between 0.87–0.96 (Quinton & Gray, 2001). To reach an estimate for the drainable porosity, we used the bulk density of the organic soil (depth 0.05–0.35 m) that ranged between 41 and 91 kg m^−3^ (Quinton & Gray, 2001). Typical values of water retained in peat soils in percentage; volume for this range of bulk densities are 25–50% (Päivänen, 1973). Assuming a porosity of 0.9 and water retention between 0.25–0.5, the drainable porosity falls in a range between 0.40 and 0.65. As these are literature‐based values, we choose the lowest bound of the range, 0.4, for the most conservative estimation, and we used this value in Equation (2) until the thaw depth reached 0.35 m. For higher thaw depths, we considered a linear decrease in porosity from 0.4 to 0.1 to simulate the influence of the mineral soil that has a lower porosity (0.43) than the organic layer. The choice of a decreasing linear estimation agreed with the assumption of heat conduction as primary means of thawing in the Stefan equation.

Deuterium excess (d‐excess = δ^2^H−8*δ^18^O) was calculated for all water samples (Dansgaard*,* 1964). Calculation of d‐excess helps to identify kinetic isotopic fractionation processes, which are typically indicative of phase change. D‐excess values <10 indicate a greater influence of endothermic kinetic isotopic fractionation processes (i.e., snow/ice melt and evaporation) and plot below the global meteoric water line (GMWL). Whereas d‐excess values equal to 10 indicate an affinity of isotopic samples to equilibrium fractionation. We used d‐excess as an additional index to distinguish between evaporated and nonevaporated streamwater sources.

To estimate the mean transit time (MTT) of the catchment, we used a transfer function to conceptualize the translation of a tracer in a catchment from input to output (Figure 2). Given the data limitations at the site, we applied an input‐weighted lumped integral convolution model (Stewart & McDonnell, 1991):

(3) $$\delta_{\text{out}}\left(t\right)=\frac{\int_{0}^{\infty }g\left(\tau \right)P\left(t-\tau \right)\delta_{\text{in}}\left(t-\tau \right)\mathrm{d\tau}}{\int_{0}^{\infty }g\left(\tau \right)P\left(t-\tau \right)\mathrm{d\tau}},$$

where τ is the TT, t is the time of interest, (t‐τ) is the time of entry to the system, δ_out_(t) is the composition at time t at output location, g(τ) is the transfer function, P(t‐τ) is the precipitation at time (t‐τ), and δ_in_(t‐τ) is the input composition at time (t‐τ). Although recent analytical approaches or modelling techniques are available to assess the time variance of the TT distribution (e.g., Ala‐aho, Soulsby, et al., 2017; Benettin et al., 2017), we had insufficient data to calibrate and independently test such models.

We assessed two different transfer functions (g(τ)), the gamma distribution and a two parallel linear reservoirs (TPLR) model, whose characteristics are summarized in Table 1. The use of each transfer function will hereafter be referred to as the gamma distribution and TPLR model. The gamma distribution is defined by a shape (α) and scale (β) parameter. The product of these parameters gives the estimate of the MTT (days). The TPLR model, proposed by Weiler, McGlynn, McGuire, and McDonnell (2003), combines fast and slow response reservoirs in the distribution function (equivalent to younger water and older water), according to a volumetric proportionality. The MTT estimated by the TPLR model is the weighted average of the reservoirs (Table 1).

**Table 1:** Description of functions, parameters and relative evaluations of mean transit time (MTT) for both tested models: gamma distribution and two parallel linear reservoirs (TPLR) model

Model	g(τ)	Parameter	MTT
Gamma	$\frac{\tau^{\alpha -1}}{\beta^{\alpha }\Gamma \left(\alpha \right)}\exp\left(-\frac{\tau }{\beta }\right)$	α = shape β = scale	*α* · *β*
TPLR	$\frac{\varphi }{\tau \left(f\right)}\exp\left(-\frac{\tau }{\tau \left(f\right)}\right)+\frac{1-\varphi }{\tau \left(s\right)}\exp\left(-\frac{\tau }{\tau \left(s\right)}\right)$	τ(f) = MTT of fast reservoir τ(s) = MTT of slow reservoir φ = volume of fast reservoir/total volume	(1 − *φ*) · *τ*(*s*) + *φ* · *τ*(*f*)

We applied three input scenarios to both models (Figure 2). The scenarios incorporated different input isotope compositions and processes: (a) measured input data (snowmelt and rainfall sampling), (b) measured input data supplemented with isotope snowmelt estimation obtained by snow isotope model simulations, and (c) Scenario 2 with the addition of estimated soil thaw water release. The isotopic composition in Scenario 3 was weighted by precipitation and thaw water release. Equation (3) was modified by replacing P by W:

(4) W=P+Th.

The input isotope composition was weighted using

(5) $$\delta_{\mathrm{inW}}=\frac{\delta_{\text{in}}P+\delta_{\mathrm{th}}\mathrm{Th}}{W},$$

where δ_th_ is the isotope composition of soil thaw water release. We calibrated the models using two different types of “output waters”: the isotope composition in streamwater at the catchment outlet and each of the soil sites. Calibration showed that of the soil sites, only the riparian soil site had reasonable fits (Nash–Sutcliffe efficiency [NSE] > 0), and therefore, further analysis was conducted using the streamwater at the catchment outlet and the riparian site soil water. Ranges of model parameters for calibration were selected in order to have the same resulting MTT range (Table 2). We used 100,000 Monte Carlo simulations for model calibration, evaluated using the NSE coefficient (Nash & Sutcliffe, 1970) and Kling–Gupta efficiency (KGE; Gupta, Kling, Yilmaz, & Martinez, 2009). The NSE was the most effective at capturing the isotopic dynamics between spring freshet and summer flows and was therefore used for our analysis. Simulations were deemed behavioural when they exceeded an NSE of 0.4. The resulting behavioural model uncertainties were evaluated applying the generalized likelihood uncertainty estimation approach (Beven & Binley, 1992). Finally, the likelihood of the TT distribution of behavioural simulations was assessed by comparing the MTT probability density function for both models.

**Table 2:** MTT model parameters used in calibration

Model	Parameter	Min	Max
Gamma	α (−)	0	2
	β (day)	0	1000
	δ_th_ (‰)	−242	−138
TPLR	τ(f) (day)	0	100
	τ(s) (day)	100	2,000
	ϕ (−)	0	1
	δ_th_ (‰)	−242	−138

*Note*. The range of thaw isotopic composition (δth) is the minimum and maximum monthly precipitation from OIPC. MTT = mean transit time; TPLR = two parallel linear reservoirs; OIPC = Online Isotope Precipitation Calculator.

## 3 RESULTS

### 3.1 Temporal dynamics in hydroclimate and stable isotopes

Air temperatures were below 0 °C until the beginning of May 2014 (Figure 3a), highest in June–August before they declined (to 2.7 °C mean monthly temperature) in September. Compared with later in the season, precipitation was low in May (on average 0.6 mm day^−1^) and increased starting in June (on average for the whole of June, 2.1 mm day^−1^; Figure 3b). Total precipitation during the study period was 190 mm. Total annual precipitation for 2014 (277 mm) was similar to the mean annual sum at Inuvik station for 1981–2010 (241 mm). However, the total precipitation during the period April–August (190 mm) was much higher than the total in that period for 1981–2010 at Inuvik (119). The snowmelt flux simulated by STARR occurred in May and had a maximum input of 21.3 mm on May 13. Soil thaw water release started on June 8 and showed a maximum of 5.1 mm day^−1^ on June 10 and gradually decreased in August (Figure 3b). Discharge showed a close link to initial rainfall and snowmelt inputs (Figure 3c). There was a large rainfall‐related event ( $\bar{Q}$ = 0.16 m^3^ s^−1^) at the start of the summer period. After the initial increase, discharge decreased to ~0.02 m^3^ s^−1^ on average during the summer months. The catchment experienced a late season peak discharge (0.12 m^3^ s^−1^) driven by a high rainfall event in August.

Rainfall composition of δ^2^H ranged from −186.7 to −105.4‰, with a mean value of −133.0‰ and standard deviation of 17.0‰ (Figure 3d; Table 3). The simulated snowmelt composition estimates (i.e., before measurements began) ranged from −221.7‰ to −164.8‰ (δ^2^H), whereas the measured snowmelt signal between June 8 and June 13 ranged from −199.6‰ and −177.6‰ (δ^2^H). The δ^2^H composition in streamwater (from the start of measurements on June 8, 2014) was much more damped than precipitation with a mean value of −157.7‰ and standard deviation of 7.3 ‰.

**Table 3:** Summary statistics of δ^2^H signatures

Water source	Mean (‰)	Max (‰)	Min (‰)	Standard deviation (‰)	No. of samples
Snowmelt sim.	−191.1	−164.8	−221.7	24.6	4
Snowmelt meas.	−191.1	−177.6	−199.6	9.9	4
Rainfall	−133	−105.4	−186.7	17	31
Stream water	−157.7	−149	−180.1	7.3	26
Riparian	−166.2	−156.3	−181.7	10.5	13

**Figure 4:**
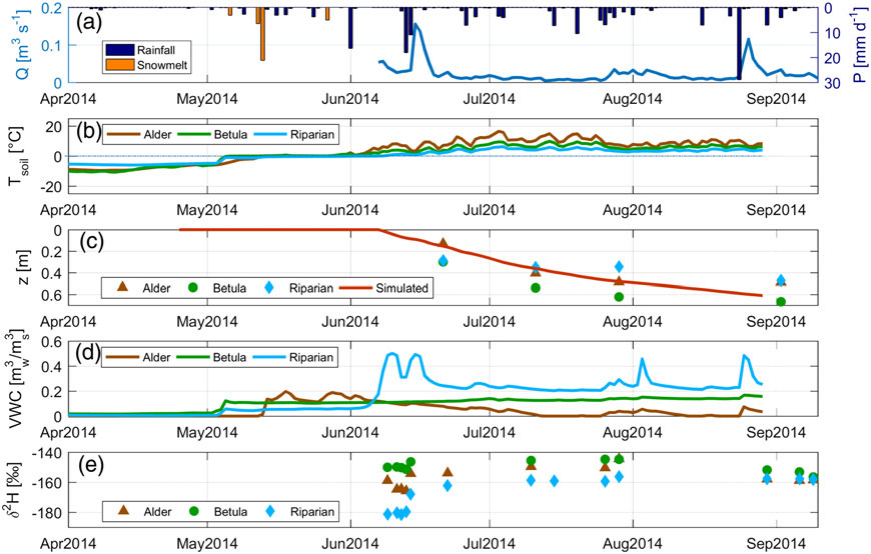
Time series data collected at the three soil profiles, including (b) soil temperature T_soil_ (°C; 5‐cm depth), (c) active layer depth measurements z (m) and simulated values, (d) volumetric water content (VWC; m^3^_w_/m^3^_s_) at 5‐cm depth, and (e) stable isotopes (δ^2^H [‰] at 10‐cm depth)

The alder and riparian soil measurement sites had highest and lowest soil temperatures (mean temperatures were 3.4 and 0.7 °C, respectively), throughout the measurement period (Figure 4b). The three soil sites all showed different active layer depth development throughout the year (Figure 4c), with riparian soils thawing latest. The active layer was deepest at the *Betula* site at the end of the season (~67 cm). The other two sites had approximately the same active layer depth at the end of the season (~45 cm). The VWC (as reported by the sensors) of all soils (Figure 4c) remained close to 0 until soil temperatures rose to 0 °C in early May, indicating the onset of soil thaw. VWC dynamics varied markedly among the three sites. Highest VWC and strongest linkages with precipitation input signals, reflected by VWC variability, occurred at the riparian site closest to the stream. The alder site showed lowest VWC corresponding to the higher temperatures and likely evaporative losses. The *Betula* site exhibited a mean VWC of ~0.1, with low variability throughout the measurement season.

Stable isotope dynamics in soil waters (Figure 4e) reflected the interplay between soil thaw water release and evapotranspiration, which in turn reflect differences in active layer development and VWC. The late thawing at the riparian site was also observed in the isotope samples having the most depleted signature during the initial sampling, <−180.0‰ and increasing to −156.3‰ at the end of June and remaining the most depleted through to September. Standard deviation at the riparian site was lower than rainfall but higher than streamwater (Table 3). δ^2^H at the *Betula* site exhibited least variability throughout the measurement period, starting at −150.0‰, and decreased during summer to ~156.5‰. δ^2^H at the alder site ranged between −165.8‰ and −144.5‰, showing some of the most enriched soil water values. Isotopic compositions (δ^2^H) for each site converged at the end of the season, though this convergence would be consistent with mixing, mainly driven by the large precipitation event at the end of August.

### 3.2 Insights into water sources and fractionation

**Figure 5:**
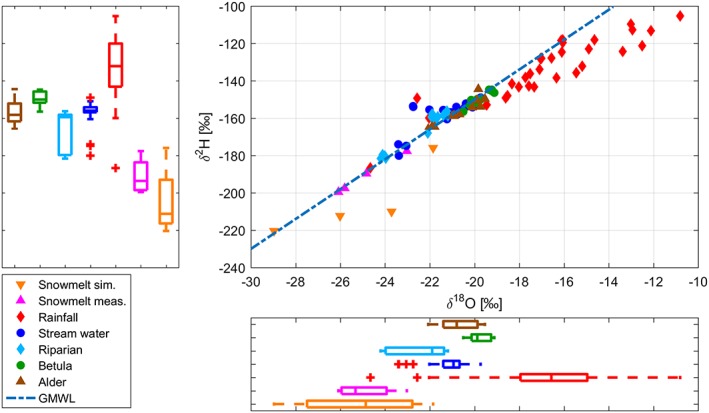
Dual‐isotope plot for different water sources during the study period May–September 2014 and overlapping the global meteoric water line (GMWL). The boxplots show the range in δ^2^H and δ^18^O stable isotopes in the different water sources

To identify differences in isotopic signatures in the different waters, the data were plotted in dual isotope space (Figure 5). Precipitation, even though only sampled for the snow‐free period, had the greatest variability. Simulated and measured snowmelt compositions were the most depleted. Stream water compositions plotted close to the GMWL, indicating no or little evaporation fractionation, with a low range between maxima and minima in both δ^2^H and δ^18^O. The signatures in the riparian soils were more depleted than streamwater and could be explained as a mix of rainfall and snowmelt but also plotting along the GMWL, indicating no or little evaporation fractionation. In contrast, soil waters at the alder and in particular, at the *Betula* site showed more enriched signatures, most likely caused by higher transpiration losses in these communities. The upper and lower quartile of the streamwater composition was generally bounded by the quartiles of the alder. Highly depleted outliers of streamwater composition were bounded by riparian soil water compositions, whereas enriched streamwater compositions were generally bounded by *Betula* soil water.

**Figure 6:**
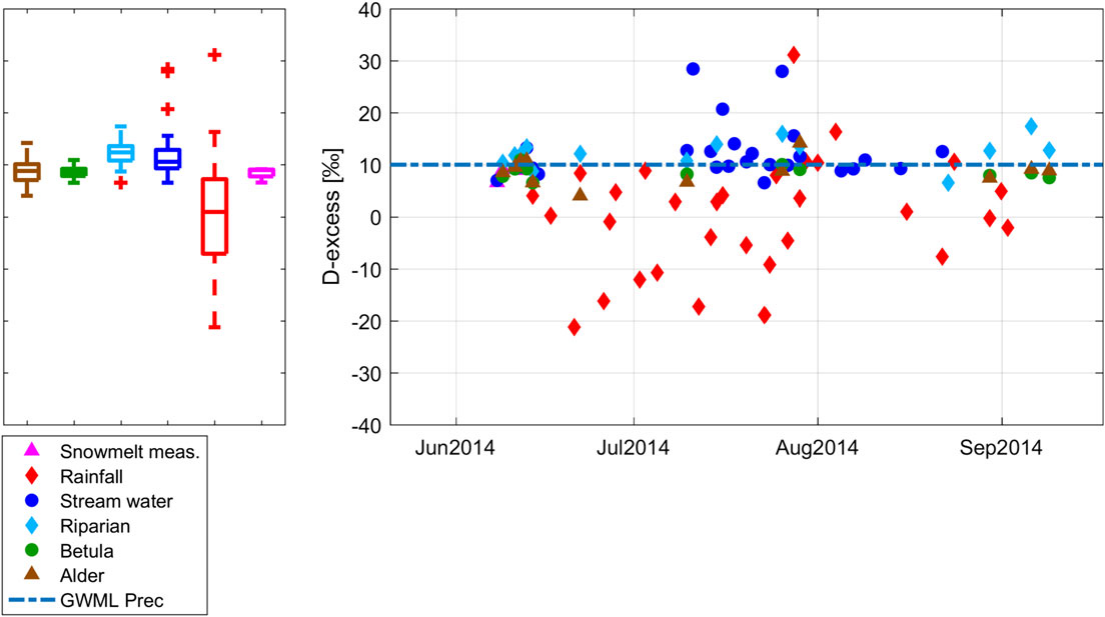
Deuterium excess (d‐excess; ‰) in snowmelt measured, rainfall, streamwater, and soil water (alder, *Betula*, and riparian sites). The reference line shows the position of precipitation on global meteoric water line (GWML), whereas the boxplots show the ranges in the different water samples. Snowmelt simulated d‐excess is not plotted as, according to model assumptions, it is located on the GWML d‐excess

D‐excess was used to explore the effects of evaporative fractionation (Figure 6). Some of the streamwater samples had very high d‐excess values (i.e., >20 ‰ on July 11, July 16, and July 23) reflecting high d‐excess values in rainfall. Late season streamwater d‐excess (August and September) did not exhibit large variability, although showed a mean less than 10‰. These lower values were more consistent with the soil water compositions of *Betula* and alder. Most of the riparian soil samples also plotted above 10‰, whereas the *Betula* and alder site soil water samples mostly fall below the d‐excess of 10‰, with the alder site showing strongest signals of evaporation (lowest average d‐excess).

### 3.3 Estimation of MTT and ages of stream and soil water

Direct comparison of the different input scenarios shown in Figure 2 was feasible through calibrated model efficiencies (NSE and KGE; Table 4). For the input Scenario 1, both the gamma distribution and TPLR model had an unsatisfactory efficiency (i.e., negative NSE), whereas modifications to the input using simulated snowmelt (Scenario 2) increased the NSE to 0.72 and 0.74 for the gamma and TPLR model, respectively. Small additional increases resulted when incorporating soil thaw: The third scenario increased the NSE to 0.79 for the gamma distribution and 0.81 for the TPLR model (Table 4). Overall, the TPLR model had only slightly higher efficiency criteria than the gamma distribution despite the additional parameter (Table 4). Similar to the NSE, the KGE for each model improved dramatically from Scenario 1 to Scenario 2, though Scenario 3 also showed small improvement. KGE values were consistently higher than NSE. However, visual inspection of the simulations revealed that the actual isotope dynamics between spring and summer were captured better using the NSE than the KGE.

**Table 4:** The best calibrated Nash–Sutcliffe efficiency (NSE) and Kling–Gupta efficiency (KGE) for the gamma and TPLR models for three different scenarios in the streamwater

Scenario	NSE gamma	NSE TPLR	KGE gamma	KGE TPLR
Only measured input data (Scenario 1)	<0	<0	0.46	0.18
+ Snowmelt simulated by STARR (Scenario 2)	0.72	0.74	0.86	0.88
++ Thaw estimation (Scenario 3)	0.79	0.81	0.89	0.90

*Note*. Efficiency less than 0 are not explicitly given (shown as <0). STARR = spatially distributed tracer‐aided rainfall–run‐off model; TPLR = two parallel linear reservoirs.

**Figure 7:**
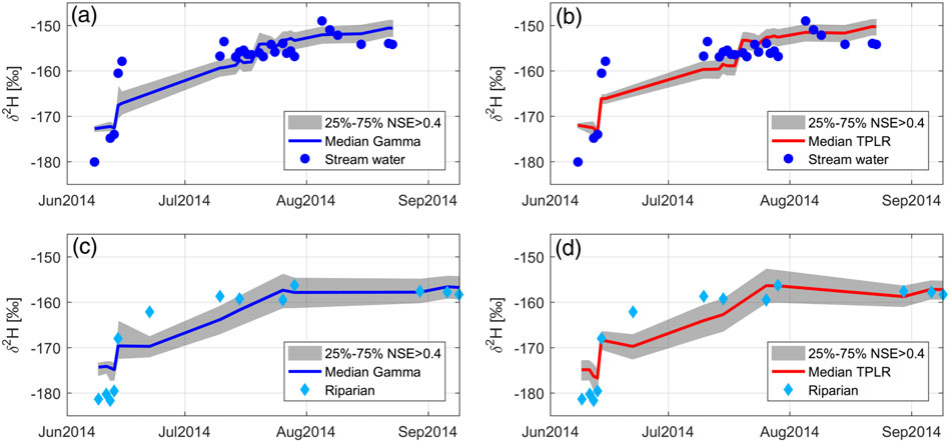
Simulated deuterium using the two models and two optimizations: (a) gamma distribution optimized on streamwater samples (blue dots), (b) two parallel linear reservoirs (TPLR) model on streamwater samples, (c) gamma distribution on soil water samples in riparian site (light blue diamonds), and (d) TPLR on soil water samples in riparian site water. Shaded areas are the 25%–75% uncertainty of behavioural simulations using generalized likelihood uncertainty estimation, whereas solid lines show the median simulations among the behavioural ones. NSE = Nash–Sutcliffe efficiency

Calibration of Scenario 3 was used to simulate isotopes in streamwater and riparian soil water and to estimate MTT within the catchment. Analysis of the calibration was conducted using behavioural parameter sets (NSE > 0.4). The median MTT of the optimized streamwater calibration was 1.7 and 1.3 years for the gamma and TPLR models, respectively (Table 5). The 25th and 75th percentiles of the estimated MTT are also summarized in Table 5 and shown as the shaded area in Figure 7. Optimizing to the riparian soil water showed slightly shorter median MTTs than streamwater estimates (1.6 and 1.2 years for the gamma distribution and TPLR model, respectively) and higher efficiencies (NSE = 0.83 and NSE = 0.85 for the gamma distribution and TPLR model, respectively). The gamma distribution showed similar ranges in the 25th and 75th percentile (MTT uncertainty) for both streamwater and riparian soil water optimization (both 2.8 years). The TPLR model had similar variation in the uncertainty range for the streamwater and riparian soil water (0.4 and 0.3 years, respectively); however the uncertainty was consistently lower than the gamma distribution.

**Table 5:** Twenty‐fifth percentile, median, and 75th percentile of the estimated behavioural MTTs (given in years) with the gamma and TPLR models using Scenario 3 (measured data, snowmelt simulation and thaw estimation)

Model	MTT 25th percentile	MTT median	MTT 75th percentile	NSE max	KGE max
Gamma (streamwater)	0.6	1.7	3.3	0.79	0.89
TPLR (streamwater)	1.2	1.3	1.6	0.81	0.90
Gamma (riparian)	0.5	1.6	3.3	0.83	0.91
TPLR (riparian)	1.2	1.2	1.5	0.85	0.91

*Note*. Also shown are the best NSE and KGE from simulation for both models. KGE = Kling–Gupta efficiency; MTT = mean transit time; NSE = Nash–Sutcliffe efficiency; TPLR = two parallel linear reservoirs.

Importantly, both models were able to capture the snowmelt depletion and isotope enrichment during the summer period in the stream and soil water optimizations (Figure 7). However, neither model captured the short temporal fluctuations in isotopic composition of the output waters (i.e., streamwater or riparian soil water). Mean uncertainty for δ^2^H was higher for the soil water (3.21‰: gamma distribution, 3.26‰: TPLR model) than for the streamwater calibration (2.51‰: gamma distribution, 2.28‰: TPLR model). The temporal change from spring to summer in isotopic compositions was much greater than the isotopic uncertainty of either model or optimization source. The relatively constrained isotopic and TT uncertainty suggests that the approach provides an appropriate first approximation to describe the general temporal response of stream and soil isotope compositions and simultaneously the MTT of the catchment.

Differences in the gamma distribution parameters were directly comparable calibrating streamwater and riparian soil water (Table 6). Similar to the differences in MTT range (Table 5), the range in parameters was comparable between streamwater and riparian soil water calibration, reduced to half of the original parameter range (Table 2). Notably, calibration showed the shape parameter (α) was estimated as more than twice the commonly calibrated catchment shape parameter of ~0.5. Similar to the gamma distribution, the parameters ranges for the TPLR model were comparable for streamwater and riparian soil water. Lastly, similar to the gamma distribution, the TPLR model showed more depleted thaw isotopic composition (δ_th_) in the calibration of the riparian soil water than the streamwater.

**Table 6:** Resulting ranges parameters (median, 25th percentile, and 75th percentile) of behavioural simulations

Model	Parameter	25th percentile	Median	75th percentile
Gamma (streamwater)	α (−)	0.7	1.2	1.6
	β (day)	292	531	768
	δ_th_ (‰)	−182	−173	−164
TPLR (streamwater)	τ(f) (day)	39.4	60.8	80.6
	τ(s) (day)	557	1024	1515
	*φ* (−)	0.21	0.46	0.72
	δ_th_ (‰)	−187	−182	−177
Gamma (riparian)	α (−)	0.7	1.2	1.6
	β (day)	267	513	759
	δ_th_ (‰)	−200	−190	−177
TPLR (riparian)	τ(f) (day)	30.7	54.6	77.5
	τ(s) (day)	572	1043	1525
	*φ* (−)	0.24	0.49	0.74
	δ_th_ (‰)	−207	−197	−187

*Note*. TPLR = two parallel linear reservoirs.

**Figure 8:**
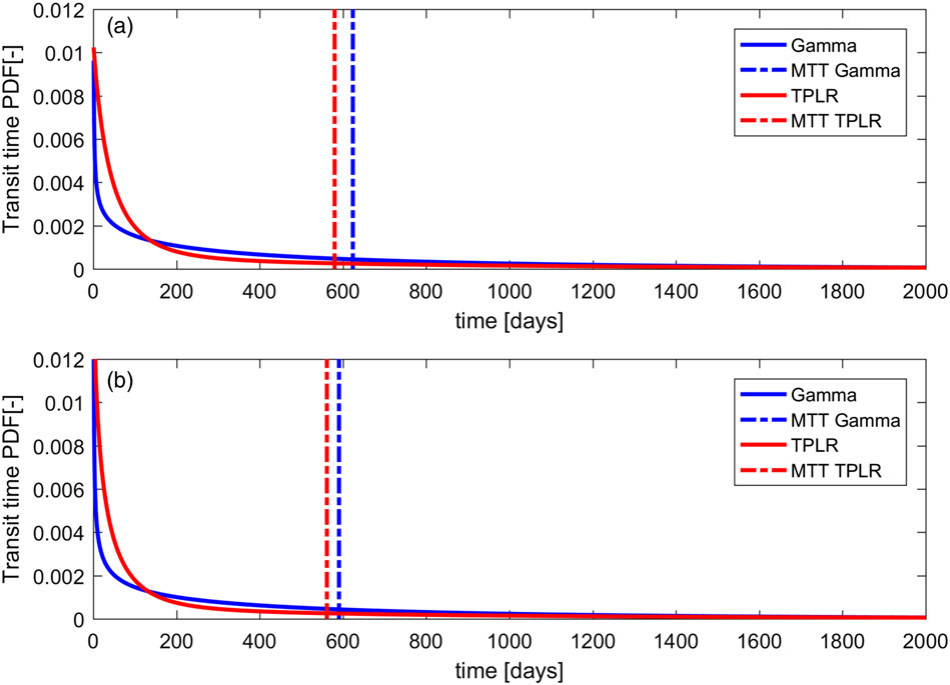
Mean transit time (MTT) probability density function of the behavioural gamma and two parallel linear reservoirs (TPLR) models over time for (a) streamwater and (b) riparian soil water. Dashed lines represent the median MTT in days for each analysed case

The comparison of the mean probability density function of the TTs in different models and output calibration shows a higher probability for younger water for the TPLR in both streamwater (Figure 8a) and soil water (Figure 8b).

## 4 DISCUSSION

### 4.1 How useful are stable isotopes for investigating the dynamics of water sources and flow paths in Arctic headwater catchments?

Over recent decades, stable isotopes have proved their utility as tools to identify and estimate hydrological sources, identify flow paths, and estimate TTs in catchments. However, tracers have been much less widely used in Arctic catchments than other environments (Tetzlaff et al., 2015). Thus, at a time of marked environmental change, stable isotopes continue to have major potential in helping to benchmark the hydrology of these sensitive northern landscapes. As a permafrost catchment, Siksik was a useful reference site to assess the challenges of applying isotope approaches in an Arctic headwater. The dominance of the snowmelt as the major driver of the most marked streamflow responses in the early spring is facilitated by the organic soils that have strong transmissivity feedback reflecting depth‐dependent porosity and permeability (Quinton & Marsh, 1999). Although the isotope signal of snowmelt is translated into streamwater (and the soils), inputs from summer rainfall and the thawing active layer release frozen water from the previous year that is more enriched than snowmelt. This allows streamwater to recover rapidly from depleting snowmelt effects (Boucher & Carey, 2010). However, the effect of the snowmelt on streamwater isotope characteristics may be more pronounced than our data imply due to the relatively late sampling caused by restricted access.

Streamflow isotopes are much less responsive to rainfall contributions than to snowmelt, suggesting that storage plays an important role in modulating run‐off generation with soil moisture deficit thresholds and soil isotope mixing (Carey et al., 2013; Carey & Debeer, 2008). Regarding storage deficits, these can potentially be explained by evapotranspiration across the catchment (Quinton & Roulet, 1998). The important role of storage at the riparian site is also supported by soil moisture dynamics pointing to a storage threshold and displacement of pre‐event water being activated during events, whereas apparent soil water deficits prevail at the *Betula* and alder sites, with the d‐excess values here also hinting at a greater evaporative influence. The riparian site had the deepest organic moss layer and a deep saturated organic horizon (>0.4‐m depth). At the *Betula* site, the organic horizon was about 0.2‐m deep and the soil isotopes were more stable at shallow depths compared with streamflow during summer with a constant VWC even despite having the deepest thaw layer.

The differing soil water storage is a potential cause of poor calibrated fits of the convolution equation at the alder and *Betula* sites, relative to the riparian site. Soil water storage has been shown to change soil thermal profiles in melt and freeze‐up conditions (discussed in Nagare, Schincariol, Quinton, & Hayashi, 2012; Hayashi, Goeller, Quinton, & Wright, 2007). Higher soil saturation may result in higher heat storage, delaying thaw and freeze‐up relative to lower soil saturation. Both the alder and *Betula* sites showed lower annual moisture, which may have expedited thaw and damped the snowmelt influence. Additionally, differences in the early thaw period soil infiltration rates at the alder, *Betula*, and riparian sites potentially result in differing recharge. The high variability of the riparian soil moisture suggests that early thaw periods (June 2014) had more open pore space relative to the alder and *Betula*. The higher open pore space may increase soil infiltration in frozen soils (Watanabe & Kugisaki, 2017). Differences in infiltration result in spatial and temporal changes in recharge (McGuire & McDonnell, 2006), though are dependent on catchment conditions that are more difficult to incorporate into traditional steady state approaches.

The seasonal separation of d‐excess of streamwater from precipitation and snowmelt suggests that more complex mixing processes occur during midsummer (July). These differences may be explained by the temporal variability of soil thaw as the active layer deepens. D‐excess values of streamwater and riparian soils are higher than either snowmelt or rainfall compositions for 2014 suggesting precipitation from the previous year rather than evaporation was the cause of deviation. Furthermore, temporal periods of higher d‐excess values in streamwater than soil waters suggest temporal changes in fractionation of thaw water. Progression freezing alters the isotopic composition of the ice while simultaneously increasing d‐excess values (Gibson & Prowse, 2002), and this may be a significant process in the autumn freeze‐up prior to the subsequent thaw season.

### 4.2 How useful are modelling frameworks to estimate isotope compositions in snowmelt and thawing soil water to supplement sparse field data?

A challenge for remote study sites like Siksik Creek is the restricted possibility for comprehensive data collection during the winter months due to the very cold climate and access limitations. Consequently, poor estimates for the snowmelt isotope input signal have been identified as a major source of uncertainty for water source or age quantification in many northern snow‐influenced environments (Peralta‐Tapia et al., 2016; Tetzlaff, Birkel, Dick, Geris, & Soulsby, 2014). In heavily snow‐influenced data‐limited environments, capturing the nonstationarity in snowmelt signals is a challenge that may be best met by data–model fusion.

It is essential to consider tracer spatial and temporal variability in tracer‐based hydrological research (e.g., hydrograph separation, TT modelling, and tracer‐aided hydrological modelling) in the Arctic (Laudon, Hemond, Krouse, & Bishop, 2002; Schmieder et al., 2016). That said, in addition to the difficulties to monitor in such remote locations, inherent large spatio‐temporal variability in snowmelt further complicates the measurements (Dahlke & Lyon, 2013; Dietermann & Weiler, 2013). The modelling approach applied here sought to overcome this data issue, incorporating both modelled precipitation and snowmelt compositions and soil water thaw rates. The incorporation of each of these modelled inputs improved the model's ability to simulate both stream and soil water compositions, in a very simplistic method compared with the known complexity established in empirical and modelling studies (Claassen & Downey, 1995; Taylor et al., 2001). For example, Feng, Taylor, Renshaw, and Kirchner (2002) investigated how melt rates affect the intensity of fractionation, with higher fractionation occurring during lower melt rates. Our parsimonious approach relates the melt fractionation to melt history rather than melt rates, though adjustment of fractionation to melt rates is possible and may be required in some snowmelt dominated catchments. Thus, modelling snowmelt isotope inputs shows potential as a means to overcome such data issues (Ala‐aho, Tetzlaff, McNamara, Laudon, & Soulsby, 2017; Ala‐aho, Tetzlaff, McNamara, Laudon, Kormos, et al., 2017).

For northern catchments, large seasonal changes in energy balance greatly affect run‐off generation (Quinton & Carey, 2008; Woo, 2012). Therefore, the estimation of input fluxes is essential for both water balance and mass balance modelling. The TT modelling approach implemented here weighted input compositions and revealed improvements in efficiency utilizing modelling soil thaw fluxes. The isotopic composition was held at a calibrated stationary value. The total soil thaw and precipitation were similar over the study period, but when thaw was active, it contributed 193 mm compared with precipitation, which was 140 mm. In the same period, run‐off was 182 mm. Furthermore, as the growing season continues to lengthen in the Arctic, the importance of water and soil thaw for vegetation is likely to increase (Jorgenson et al., 2013).

### 4.3 Estimating water ages and TT in data sparse Arctic regions

Challenges of assessing water balance, and thereby water age and TT, in Arctic watersheds are driven by site access and data limitations (Bring et al., 2016; Lique, Holland, Dibike, Lawrence, & Screen, 2016). Data availability and temporal and spatial variability introduce a broader question: Do TT models work in the Arctic? If so, which models are useful and what approach is most useful? In our study, TT estimations were restricted to first approximations during the spring and summer due to a short data collection period. Through incremental integration of processes used to derive the input data, we demonstrated how modelling methods can be used to supplement the isotope field data, particularly those that are difficult to collect during winter conditions. The model–data fusion used in this study facilitated a more viable input for the TT modelling, where all relevant water sources (snowmelt, soil thaw, and rainfall) are considered. Increasingly complex characterization of the model input signal resulted in a step‐wise improvement in model fit. Importantly, both the gamma distribution and the TPLR model were able to capture the snowmelt depletion and isotope enrichment during the summer period in the stream and soil water optimizations (Figure 7). However, neither model captured the short temporal fluctuations in isotopic composition of the output waters (i.e., streamwater or riparian soil water). The resulting MTTs were ~1.5 years, thus integrating the short TTs of the hydrologically dominant snowmelt and longer TTs of summer and fall active layer storage. The fast isotope dynamics in the spring were not fully captured by the TT modelling, due to assumed stationary storage, a valid assumption in temperate climates. However, in permafrost environments, thawing results in storage changes during spring and summer resulting in longer lag times between event and stream response, though this change in storage is predictable (Carey & Debeer, 2008; Carey & Woo, 2001; Streletskiy et al., 2015). Estimates of streamwater and soil water ages indicated essentially similar but slightly shorter TT distributions for soil water. These similarities were reasonable given the strong relationship of stream discharge and the soil moisture responsiveness, in addition to the proximity of the riparian soils to the stream. However, as early freshet isotopic stream and soil compositions were unavailable for calibration, the MTT of each simulation may be overestimated due to underestimating the contribution of young water during the rising limb of the freshet. Even when measured, rapid initial response when melt season starts and when storage is very low (mainly depression storage) may be particularly difficult to capture due to heterogeneity (Fuss, Driscoll, Green, & Groffman, 2016). Nevertheless, a key result of the paper is that inputs to the catchment from snowmelt, and soil thaw have a considerable impact on our understanding of how water ages and TTs evolve.

**Figure 9:**
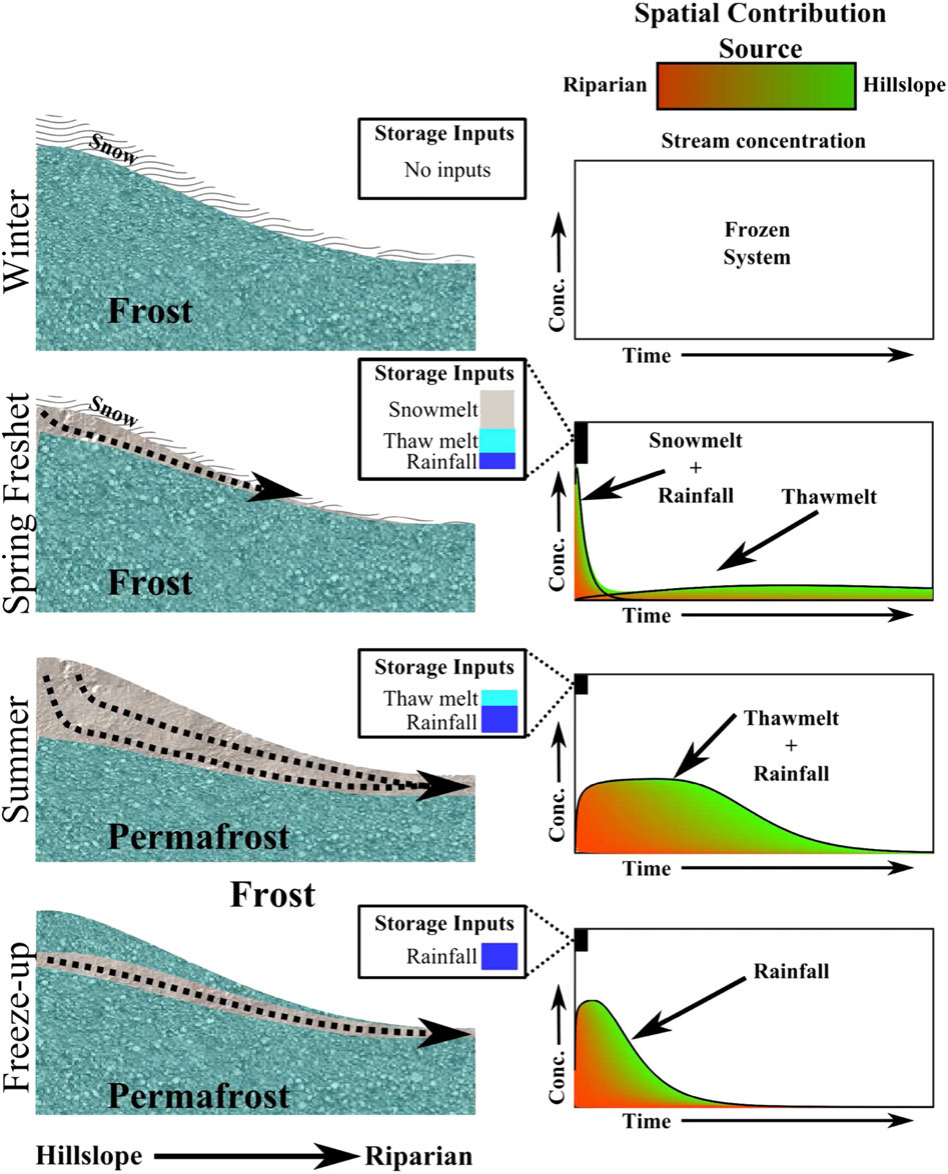
Conceptual diagram of temporal changes in transit time distributions in Arctic environments. Three temporal periods are shown: (a) winter, (b) freshet, and (c) summer, with spatial contribution to the stream (i–v, closest to furthest). Additionally shown is the cumulative spatial stream contribution of input types (i.e., snowmelt, rainfall, and thaw melt)

Despite TT uncertainties due to data limitations, these preliminary estimates allow us to conceptualize how snowmelt and soil thaw and summer precipitation likely interact with catchment sources to affect resulting TTs (Figure 9). The graphic shows how early spring TTs are short, dominated by snowmelt and limited catchment storage when soils are predominantly frozen, although some infiltration of meltwater occurs (Zhang, Carey, Quinton, Janowicz, & Flerchinger, 2010). Furthermore, the spring is dominated by high influxes of snowmelt, when almost 60% of the annual precipitation enters storage within a short period (approximately 1 month). Significant and increasing mixing with water in the active layer during the summer results in more damped streamwater variation, even in large precipitation events, giving the longer MTTs. As the summer progresses, TT model application may become more feasible when the contributing volume is increasingly stable; however, these equations may not capture peak events if inputs are large relative to the available storage (e.g., fill‐and‐spill mechanisms; Spence & Woo, 2003; Hrachowitz et al., 2016). However, summer TTs and run‐off are highly dependent on the annual development of the active layer and the depth to permafrost (Wright, Hayashi, & Quinton, 2009). Multiple factors may influence the active layer storage in each year, including precipitation, snowmelt timing, and soil temperature (Wright, Quinton, & Hayashi, 2008). Cooler summers or late freshet periods reduce active layer development whereas warmer summers and longer growing seasons expand the active layer, increasing the MTTs due to greater mixing (Lyon et al., 2010). The freeze‐up period results in a restriction of subsurface flow paths due to thermal ice growth from both the surface downward and the permafrost upward. The growth of soil ice is controlled by multiple factors including summer temperatures, active layer thaw progression, and soil saturation (Hayashi et al., 2007; Nagare et al., 2012). The effect of saturation on heat storage and freeze‐up may result in spatial variability particularly in the saturated riparian zone. Faster freeze‐up upslope and slower freeze‐up near stream may combine to decreasing catchment TTs. These temporal changes need to be considered to estimate long‐term changes in water flow paths. Thus, traditional steady state approaches are limited when the storage changes are so dramatic.

### 4.4 Further challenges of assessing water ages in Arctic catchments and possible ways forward

Quantifying the actual age of thaw and snow water is difficult, as spring thaw water existed in storage prior to freezing in fall, snow accumulates throughout the winter with various ages, and refreezing affects the isotopic composition of each. Thus, characterizing the TT distribution requires integration of young waters as most (if not all) snowmelt water input has an age of 0 at time (t‐t_i_), but this displaces soil thaw and permafrost thaw which is at least one season older.

More recent spatially distributed modelling approaches with tracer‐aided models (e.g., Ala‐aho, Tetzlaff, McNamara, Laudon, & Soulsby, 2017; van Huijgevoort et al., 2016) offer potential for greater process insights of time variance for permafrost systems where spatially distributed processes are complex. But even here, fundamental issues affecting tracer inputs relating to snow and soil properties, which ultimately relate to snow accumulation, melt, and soil thaw, are serious challenges. In particular, aspect and wind‐blown snow have a large effect on snow accumulation in this region (e.g., Quinton & Carey, 2008). Pomeroy, Marsh, and Gray (1997) applied a snow‐blowing model in Trail Valley Creek using a digital elevation model, which has promise for coupling with spatially distributed isotope‐based snowmelt models (Ala‐aho, Tetzlaff, McNamara, Laudon, & Soulsby, 2017). However, even then the spatial heterogeneity in soil thaw and active layer development would need to be considered. Furthermore, data are needed to calibrate these models. In the absense of measured field data, recent developments in remote sensing may provide new methods of data assimilation. Such methods have already been used in modelling snowmelt‐dominated alpine catchments (Bach, Braun, Lampart, & Mauser, 2003) and estimating snowmelt in Arctic tundra (Kepski et al., 2017).

Notwithstanding the logistical challenges of working in Arctic ecosystems, the importance of these extensive areas vulnerable to climate and other environmental change prioritize them as important locations for hydrological research. It will be difficult, if not impossible, to assess the implications of environmental change in nonstationary times as little is known about water stores, flow pathways and residence times. Data availability and collection is likely to remain a challenge, especially in times of dimishing field work focus in hydrology in general (Burt & McDonnell, 2015) and trends in reduced funding for catchment studies (Laudon et al., 2017; Tetzlaff, Carey, McNamara, Laudon, & Soulsby, 2017). Although field campaigns will be essential to data collection, modelling frameworks as applied here can be used to increase process understanding. Furthermore, the critical role of landscape‐scale hydrology in biogeochemical processes (e.g., carbon cycling and net greenhouse gas fluxes; the transport and fate of environmental contaminants) and vegetation dynamics is also becoming increasingly apparent in permafrost catchments (Bring et al., 2016; Elmendorf et al., 2012; Martin, Jeffers, Petrokofsky, Myers‐Smith, & Macias‐Fauria, 2017; Street et al., 2016; Wrona et al., 2016); these critical linkages between hydrology, ecosystem dynamics, and the broader earth system, together with the logistical and practical challenges of data collection in the Arctic, make robust hydrological modelling imperative in this rapidly changing region.

## 5 CONCLUSION

Arctic catchments are highly sensitive to temporal changes of precipitation and temperature. They are characterized by high contributions of snowmelt, limited water storage in soils in early spring, and an important role of soil thaw in run‐off generation. Our step‐wise approach with data–model fusion included the temporal contributions of rainfall, snowmelt, and soil thaw to streamwater and soil water in a data‐sparse Arctic catchment. The models developed in this study were able to capture the observed snowmelt depletion and isotope enrichment during the summer for the stream and soil water but were not able to capture short temporal fluctuations in isotopic composition of the output waters. Increasing complexity with the addition of all inputs yielded the best model results and helped inform on the importance of each season and input. MTTs of stream and soil waters were a mixture of rapid response snowmelt during the freshet when storage was small, and slow response during the summer months when soil thaw has progressed. Stream water isotopic variation was restrained during summer and exhibited longer TTs (~1.5 years) as a result of increased mixing with water in the active layer. We also showed that isotope mixing, tracer‐aided models need to incorporate the presence of “old” water (water stored from the previous season and released) during spring snowmelt.

Our findings help to improve the understanding of processes essential to estimating catchment water ages and flow paths in Arctic catchments. The data limitations due to remote and difficult access, particularly during the winter, create additional challenges beyond the already complex cold‐weather processes. As the contribution of each source to catchment storage and run‐off changes due to rapid environmental changes, continued evaluation of Arctic catchments via measurement and modelling is essential to predict long‐term changes. The tracer‐aided results presented in this study provide a baseline for an improved understanding of temporal dynamics and source inputs of water mixing in small Arctic catchments.
